# Resveratrol Protects against Methylglyoxal-Induced Hyperglycemia and Pancreatic Damage *In Vivo*

**DOI:** 10.3390/nu7042850

**Published:** 2015-04-15

**Authors:** An-Sheng Cheng, Yu-Hsiang Cheng, Chi-Ying Lee, Chin-Yuan Chung, Wen-Chang Chang

**Affiliations:** 1 Department of Medicinal Plant Development, Yupintang Traditional Chinese Medicine Foundation, 2F., No. 398, Zengzi Rd., Zuoying District, Kaohsiung City 813, Taiwan; E-Mails: d99628009@ntu.edu.tw (A.-S.C.); saminmon@gmail.com (C.-Y.L.); m0604681@gmail.com (C.-Y.C.); 2 Department of Science, University of Auckland, 23 Symonds Street, Auckland 1142, New Zealand; E-Mail: yche556@aucklanduni.ac.nz

**Keywords:** resveratrol, methylglyoxal, nuclear factor erythroid 2-related factor 2, retinoic acid, diabetes

## Abstract

Methylglyoxal (MG) has been found to cause inflammation and insulin resistance *in vitro* and *in vivo* in recent studies. Resveratrol has been proposed as an effective treatment that helps lower the risk of developing complications of diabetes. To study the significance of glycosylation-related stress on the pathology of diabetes, the effects of resveratrol were examined in a mouse model of diabetes induced by MG. Resveratrol was given via oral gavage in MG-treated mice, and diabetes-related tests and markers were assessed using biochemical and immunohistochemical analyses. Treatment with resveratrol markedly improved blood glucose level from the oral glucose tolerance test and promoted nuclear factor erythroid 2-related factor-2 (Nrf2) phosphorylation (*p* < 0.05) in the pancreas of MG-treated mice. However, these effects were abolished by retinoic acid, Nrf2 inhibitor, in resveratrol and retinoic acid-treated and MG-induced mice. These findings support that resveratrol may be useful in the treatment of type-2 diabetes by protecting against pancreatic cell dysfunction.

## 1. Introduction

Hyperglycemia can initiate the production of advanced glycation end products (AGEs) by the non-enzymatic reaction of carbohydrates and proteins [1,2]. AGEs have been reported to induce the formation of free radicals and other reactive intermediates, triggering inflammatory response and the progression of diabetes [3]. Methylglyoxal (MG) is a highly-reactive dicarbonyl metabolite of glucose metabolism [4] and α precursor of AGEs that can lead to the development of diabetes. Recently, MG has been found to cause impairment of pancreatic cell function and suppression of insulin secretion in Sprague-Dawley rats [5,6].

Nuclear factor erythroid 2-related factor 2 (Nrf2) is an antioxidant-responsive element (ARE)-associated transcriptional factor that elevates heme oxygenase-1 and glutamate-cysteine ligase expression [7,8]. Activation of Nrf2 also induces glyoxalase-1 expression, which promotes MG to form d-lactic acid [9]. A prior study has reported an association between oxidative stress and cell damage [10]. However, a higher level of MG (200 ppm) was found in patients with diabetes compared to people without diabetes (5 ppm) [11]. It has been reported that coffee, cream and cake could elevate the MG level in serum, with coffee containing approximately 230 μM of MG [12] and honey containing 5 mg/g of MG [13]. In Spain, people consume 216 μg of MG in cookies per day [14]. Moreover, wine and beer contain 1.6 and 1.0 μg/mL of MG, respectively [15,16].

Several experimental models among various studies have demonstrated that dietary phytochemicals, such as resveratrol, have exhibited significant bioactivities [17,18]. Resveratrol is a stilbene-type phytoalexin that is found in a wide variety of plants and fruits, such as legumes, grapes and berries, and has many reported health benefits, including anti-diabetic properties [19,20,21,22,23]. It acts through multiple pathways, including AMP-activated protein kinase (AMPK), sirtuin 1 (SIRT1) and nuclear factor erythroid 2-related factor 2 (Nrf2), to protect against pancreatic β-cell dysfunction and improve insulin sensitivity [24,25,26,27,28]. It has also been found to ameliorate diabetes by inhibiting inflammatory response, poly(ADP-ribose) polymerase and phosphodiesterase (PDE) activities [29,30].

To date, various pharmacological studies have utilized resveratrol mainly as an oral supplement to treat diabetic patients [31,32], which come in the form of tablets and capsules and may vary in terms of doses [33,34]. Previous studies demonstrated that, regardless of the difference between the doses, resveratrol may still exhibit antidiabetic effects on patients [31,32]. The administering of higher doses lead to the activation of AMPK (SIRT1-independent), whereas lower doses (<0.5 g/person) promote the activation of SIRT1, as well as improving insulin sensitivity and glucose levels, while only inducing moderate reversible side effects [31,35]. Despite the positive aspects exhibited by the consumption of resveratrol, past studies have identified a clear need for the improvement of resveratrol bioavailability [34]. This is attributed to the involvement of sulfation and glucuronidation of resveratrol metabolism in humans, where it has been noted that a quick phase II conjugation towards the formation of the two metabolites explains the low bioavailability (oral) of resveratrol [36]. Despite this, there has been increasing scientific evidence promoting the positive bioactivities of resveratrol, as well as identifying it as a promising supplement and nutraceutical for diabetes treatment [37].

Retinoic acid (RA) has been reported to suppress the activation of Nrf2 mediated by RA receptor-α (RARα) [38]. Our previous study has shown that resveratrol effectively attenuated insulin resistance induced by MG in Hep G2 cells [39]. However, the anti-inflammatory and antidiabetic effects of resveratrol have not been examined in mice with MG-induced diabetes. The purpose of this study was to evaluate the anti-diabetic effect of resveratrol, with or without the addition of RA, on insulin resistance in MG-treated mice.

## 2. Materials and Methods

### 2.1. Reagents

Resveratrol, retinoic acid (RA), MG and pioglitazone were purchased from Sigma Chemical Co. (St. Louis, MO, USA). The protein concentration was determined using a standard commercial kit (Bio-Rad Laboratories, Hercules, CA, USA). Anti-p-Nrf2 antibody was purchased from Bioss (Woburn, MA, USA). Anti-insulin antibody and IRS-1 (tyrosine phosphorylation) enzyme-linked immunosorbent assay (ELISA) kits were purchased from Cell Signaling Technology (Beverly, MA, USA).

### 2.2. Diabetes Induction

Male Balb/C mice (4 weeks old, *n* = 30) were obtained from the National Laboratory Animal Breeding and Research Center (Taipei, Taiwan) and acclimatized for 1 week prior to use. The experimental protocol complied with the guidelines described in the “Animal Protection Law”, amended on 17 January 2001 (Hua-Zong-(1)-Yi-Tzi-9000007530, Council of Agriculture, Executive Yuan, Taiwan, ROC). The animals were provided with food and water *ad libitum* and subjected to a 12-h light/dark cycle at 25 °C, with a relative humidity of 60%. Mice were divided at random into the following five treatment groups: (1) control; (2) MG (1% in water, oral administration); (3) MG + pioglitazone (10 mg/kg, oral administration); (4) MG + resveratrol (10 mg/kg; oral administration); and (5) MG + resveratrol + RA (10 mg/kg, intraperitoneal injection). These treatments were administered daily for 12 weeks.

### 2.3. Blood Sample Collection

Blood samples were collected and allowed to clot for 30 min at room temperature and then centrifuged at 3000× *g* for 20 min to obtain the serum, which was stored at −80 °C before use.

### 2.4. Oral Glucose Tolerance Test and Insulin Tolerance Test

The oral glucose tolerance test (OGTT) was performed after an overnight fast. A basal blood sample was taken, followed by oral administration of glucose (2 g/kg). Blood was collected for up to 120 min, and blood glucose was determined using a glucose assay kit (BioAssay Systems, Hayward, CA, USA). For the insulin tolerance test (ITT), mice were given an intraperitoneal injection of insulin (0.5 U/kg) after an overnight fast, followed by blood glucose determination for 45 min.

### 2.5. Serum Insulin, Hepatic Inflammatory Factors and Tyr-Phosphorylated Insulin Receptor Substrate-1 Protein Expression Assay

Insulin level was measured using an ELISA assay kit (Mercodia, Winston Salem, NC, USA). Cytokines (tumour necrosis factor α (TNF-α) and interleukin 1β (IL-1β)) were measured using ELISA kits purchased from Peprotech (Rocky Hill, NJ, USA). Tyr-phosphorylated insulin receptor substrate-1 (IRS-Tyr) was measured using an ELISA assay kit (Cell Signaling Technology, Beverly, MA, USA). Analyses were performed following the supplier’s protocols.

### 2.6. Homeostasis Model Assessment-Insulin Resistance

The homeostasis model assessment for insulin resistance (HOMA-IR) was calculated via the following equation: fasting serum insulin (mU/L) × fasting glucose (mmol/L)/22.5 [40].

### 2.7. Immunohistochemistry Stain

Frozen 6-μm sections were cut on a cryostat and thaw-mounted onto silane-coated slides. The sections were incubated with 3% H_2_O_2_ for 20 min to quench endogenous peroxidase activity. After being rinsed twice with phosphate-buffered saline (PBS), the sections were incubated with skim milk (5%) for 1 h, followed by incubation with anti-p-Nrf2 (1:100) for 12 h. After washing in PBS, the sections were incubated with secondary antibody (1:200) in PBS for 1 h. After two more rinses with PBS, the immunoreactive signal was visualized by incubation with 3,3’-diamino benzidine tetrahydrochloride for 10 min. The sections were counter-stained with hematoxylin.

### 2.8. Statistical Analysis

Experimental results were expressed as the mean (standard deviation, SD) of three parallel measurements. The results were subjected to one-way analysis of variance (ANOVA) to determine the statistical significance of differences between sample means. *p* ≤ 0.05 was considered statistically significant.

## 3. Results

### 3.1. The Anti-Diabetic Effects of Resveratrol in MG-Treated Mice

**Figure 1:**
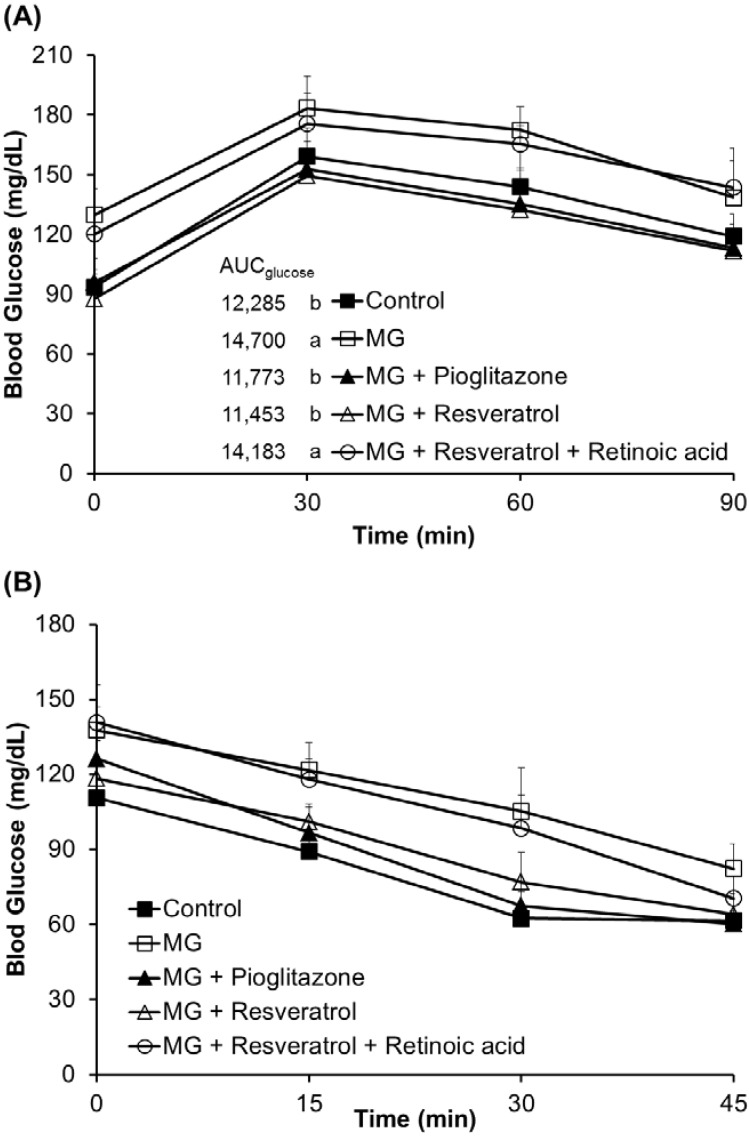
The effects of resveratrol on the (**A**) oral glucose tolerance test (OGTT) and the (**B**) insulin tolerance test (ITT). The data shown represent the mean ± SD (*n* = 6). (a,b) indicate a statistically-significant difference at *p* < 0.05. MG: methylglyoxal.

The fasting serum glucose levels were significantly higher in rats fed with MG (129.8 ± 13.1 mg/dL) for 12 weeks prior to supplementation with resveratrol, relative to those in rats fed a normal diet (93.5 ± 6.7 mg/dL) (*p* < 0.05). This indicates the induction of hyperglycemia in rats. Figure 1A shows the effect of resveratrol on serum glucose levels during OGTT in MG-fed rats. There were significant differences in initial serum glucose levels among those groups. In addition, MG-treated diabetic rats given resveratrol had significantly reduced serum glucose levels (*p* < 0.05) (Figure 1A). The integrated area under the glucose curve of OGTT in MG-treated diabetic rats showed a significant increase in serum glucose *versus* the other groups (*p* < 0.05) (Figure 1A). ITT was significantly lower in MG-fed rats given 10 mg/kg resveratrol, 10 mg/kg pioglitazone and normal diet than in the MG group (Figure 1B).

**Figure 2:**
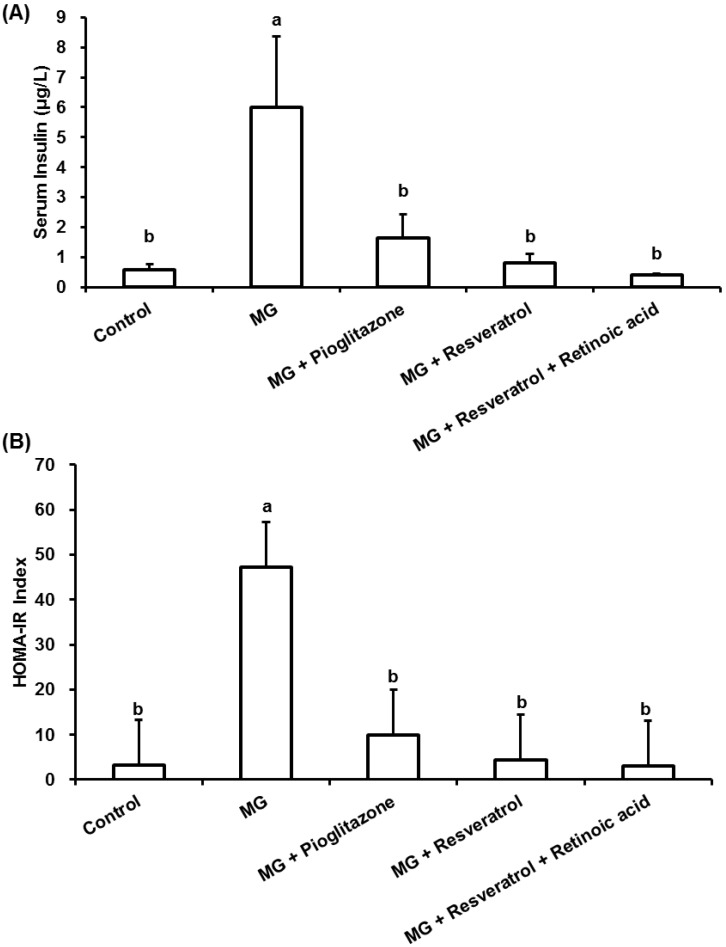
The anti-diabetic effects of resveratrol on (**A**) serum insulin and (**B**) homeostasis model assessment-insulin resistance (HOMA-IR) in methylglyoxal-treated Balb/C mice. The data shown represent the mean ± SD (*n* = 6). (a,b) indicate a statistically-significant difference at *p* < 0.05.

MG-fed rats showed a significant increase in the level of insulin and the value of the HOMA-IR index in comparison to normal rats (*p* < 0.05) (Figure 2). Administration of resveratrol improved insulin resistance as demonstrated by the reduced 86.2% insulin levels in this group (Figure 2A) in comparison to the MG group. Administration of resveratrol led to a significant reduction in the value of HOMA-IR index of rats that was elevated by MG feeding. There was no significant difference between the resveratrol-treated group and the normal group, in which the HOMA-IR index value was also lower than the MG-treated rats alone (Figure 2B).

### 3.2. Anti-Inflammatory Effects of Resveratrol

**Figure 3:**
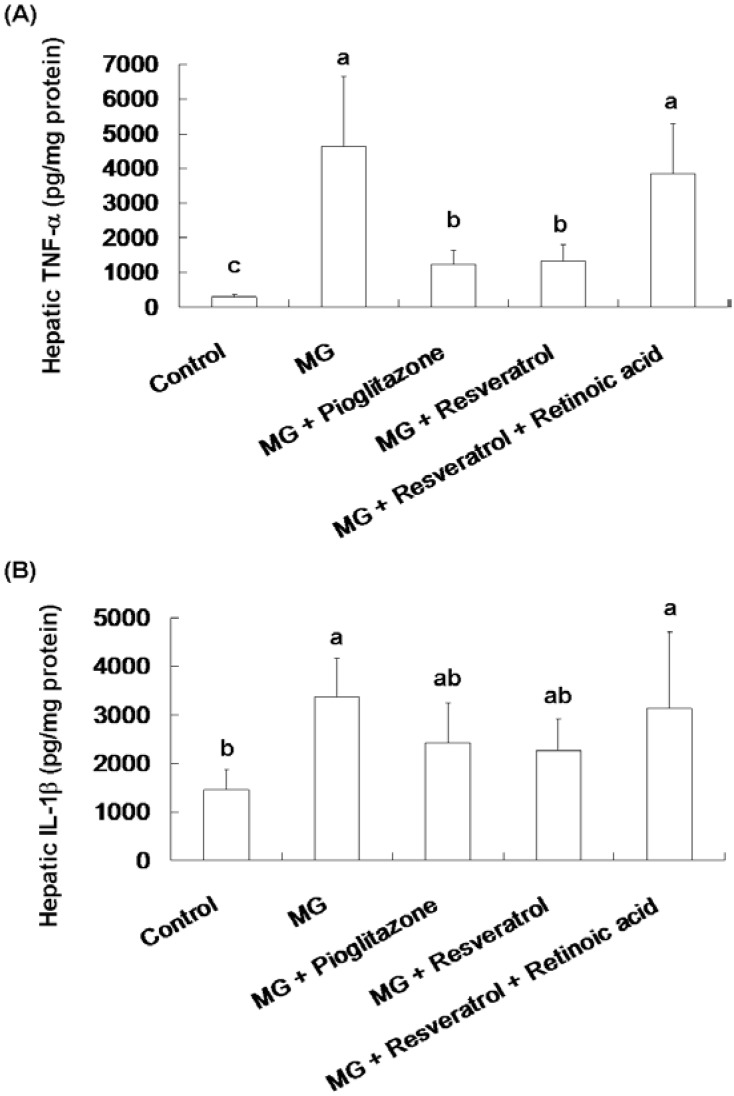
The suppression of inflammatory cytokine levels in the livers of methylglyoxal-treated mice treated with resveratrol. (**A**) Hepatic TNF-α and (**B**) Hepatic IL-1β levels of methylglyoxal treated mice. The data shown represent the mean ± SD (*n* = 6). (a–c) indicate a statistically-significant difference at *p* < 0.05.

Figure 3 shows the effect of resveratrol on hepatic TNF-α and IL-1β contents in rats. Significantly higher hepatic TNF-α and IL-1β contents were observed in the MG group compared to the normal group, 4641.8 ± 2094.3 pg/mg protein *vs.* 283.5 ± 86.4 pg/mg protein and 3369.2 ± 790 pg/mg protein *vs.* 1462.9 ± 426.3 pg/mg protein, respectively (*p* < 0.05). MG-fed rats given pioglitazone and resveratrol had significantly reduced TNF-α contents of 73.6% and 71.3%, respectively (Figure 3A). Co-administration with resveratrol and retinoic acid did not alter the TNF-α and IL-1β of rats (Figure 3).

### 3.3. Resveratrol-Mediated Elevation of Liver Tyr-Phosphorylated IRS-1 Levels

**Figure 4:**
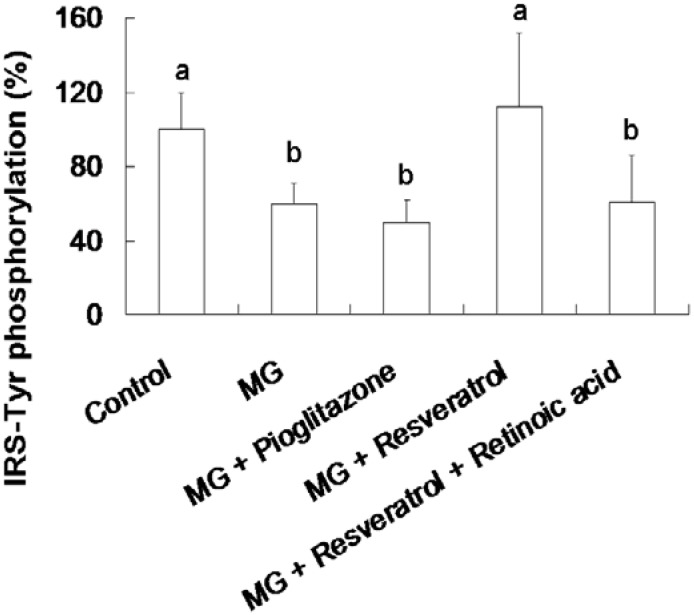
Elevation of hepatic insulin receptor substrate 1 (IRS-1) tyrosine (Tyr) phosphorylation in methylglyoxal-treated mice treated by resveratrol. The data shown represent the mean ± SD (*n* = 6). (a,b) indicate a statistically-significant difference at *p* < 0.05.

Figure 4 shows the resveratrol-mediated elevation of liver Tyr-phosphorylated IRS-1 levels in rats. The animal group fed with the MG-supplemented diet showed a 39.5% reduction in Tyr-phosphorylated insulin receptor substrate-1 (IRS-Tyr) expression in the liver compared to the control group (*p* < 0.05). Resveratrol (10 mg/kg body weight) enhanced IRS-Tyr expression in MG-fed rat liver by 85.5% of the rats fed with MG alone (*p* < 0.05). Moreover, there is no significant difference in the hepatic level of IRS-Tyr protein expressions in MG-administrated rats with added with pioglitazone or resveratrol and retinoic acid together (Figure 4).

### 3.4. IHC Stain for Pancreatic Insulin and p-Nrf2

**Figure 5:**
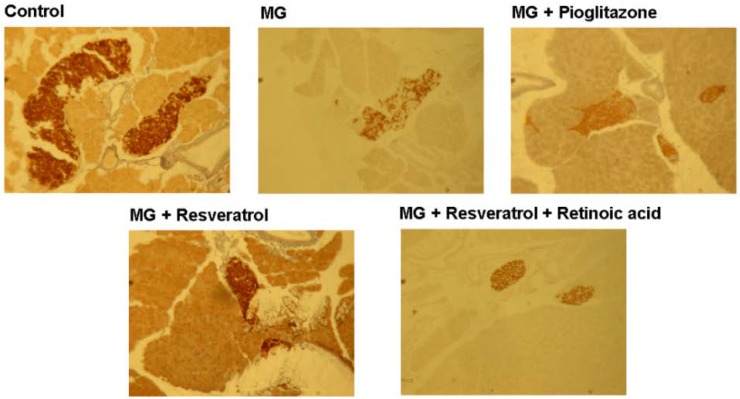

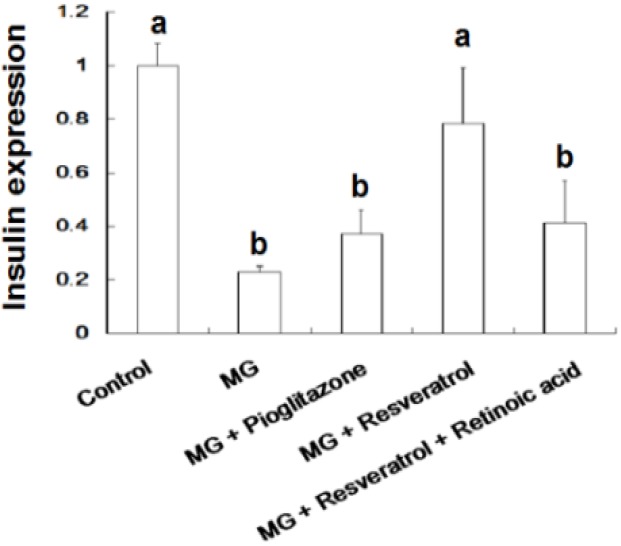
Immunohistochemical visualization of pancreatic insulin in methylglyoxal-treated mice treated with resveratrol (*n* = 6). The bars represent the pancreatic cellular insulin content of different treatment groups. (a,b) indicate a statistically-significant difference at *p* < 0.05.

**Figure 6:**
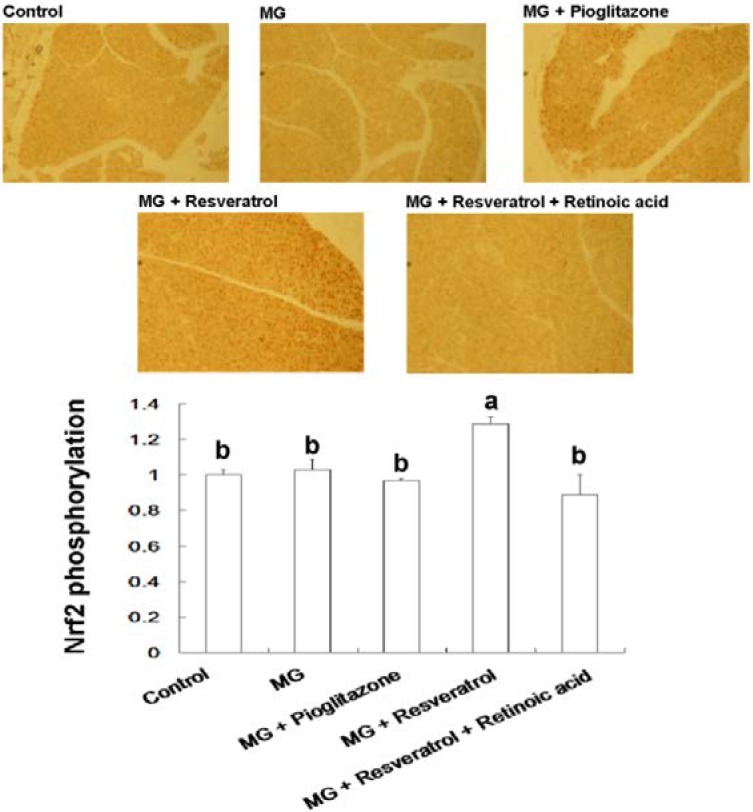
Immunohistochemical visualization of pancreatic p-Nrf2 in methylglyoxal-treated mice with resveratrol (*n* = 6). The bars show the pancreatic cellular content of p-Nrf2 of different treatment groups. (a,b) indicate a statistically-significant difference at *p* < 0.05.

Figure 5 and Figure 6 show the immunohistochemistry (IHC) stain for pancreatic insulin and p-Nrf2 protein expression in rats. Our data show that the pancreatic contents of insulin and p-Nrf2 protein expression were decreased in MG-administrated rats alone (*p* < 0.05). The pancreatic level of insulin and p-Nrf2 protein expressions were increased in MG-administrated rats with added resveratrol (*p* < 0.05). However, there was no significant difference in the pancreatic level of insulin and p-Nrf2 protein expressions among MG-administrated rats with added pioglitazone or resveratrol and retinoic acid together, indicating the occurrence of the imbalance of redox after the administration of MG (Figure 5 and Figure 6).

## 4. Discussion

After 12 weeks of treatment, the effects of resveratrol on OGTT and ITT in Balb/C mice were investigated. As shown in Figure 1A, MG treatment clearly resulted in hyperglycemia in the OGTT, while pioglitazone (an anti-diabetic drug) and resveratrol both reduced blood glucose (0–120 min). However, the hypoglycaemic effect of resveratrol was attenuated by RA administration. In addition, ITT was used to evaluate liver, muscle and adipose tissue insulin sensitivity. These results (Figure 1B) suggested that administration of resveratrol and pioglitazone with MG lowered blood glucose in the ITT, as compared with animals administered MG only. This insulin-sensitizing effect of resveratrol was also attenuated by RA treatment. After sacrifice of the mice, determination of serum insulin and blood glucose (Figure 2) showed that MG induced marked hyperinsulinemia and hyperglycemia and indicated that these diabetic features were improved by co-administration of pioglitazone or resveratrol. We found that pioglitazone and resveratrol significantly suppressed liver TNF-α and IL-1β levels in Balb/C mice with MG-induced diabetes. However, the inhibitory effects of resveratrol on inflammatory cytokines were abolished by RA treatment (Figure 3). These findings revealed that Nrf2 activation was involved in resveratrol’s anti-inflammatory effects.

Type-2 diabetes (T2-D) is a chronic disease of carbohydrate metabolism caused by a deficiency in insulin secretion or ineffective insulin action. Medicinal plants provide common alternative treatments for T2-D in many parts of the world [37]. Impaired insulin signaling leads to reduced glucose uptake and glycogen synthesis, eventually causing insulin resistance in hepatocytes [41]. This loss of the ability of insulin to trigger downstream metabolic actions in the liver is defined as hepatic insulin resistance [42]. In theory, Tyr phosphorylation of IRS may activate protein kinase B (PKB)/Akt signaling, which promotes GLUT translocation to the membrane, thereby improving insulin sensitivity [43]. TNF-α reduces insulin-dependent signal transduction through a mechanism involving insulin receptor (IR) and IRS down-regulation, inhibition of IR and IRS Tyr phosphorylation and increased serine serine/threonine phosphorylation of IRS. Furthermore, protein Tyr phosphatase1B (PTP1B) has been reported to inhibit glucose transporter (GLUT) expression, through decreasing IRS Tyr phosphorylation [44]. When insulin binds to the IR α-subunit, the intracellular β-subunit becomes activated by phosphorylation of specific Tyr residues, which, in turn, causes binding and activation (Tyr phosphorylation) of IRS. This is the most important receptor substrate in cells that respond to insulin, causing Akt phosphorylation and GLUT translocation [45]. Studies have reported that resveratrol can improve metabolic disorders through activation of Sirtuin 1 (SIRT1) and Nrf2 expression in high fat diet-induced obese mice [46,47,48]. The mechanism by which resveratrol activates SIRT1 is not fully understood. However, it has been proposed that activation of AMPK, along with inhibition of phosphodiesterase 4 and elevation of cAMP in the cell, is essential for resveratrol to allosterically control SIRT1 expression [47]. A recent study also demonstrated the link between SIRT1 and the metabolic health benefits of resveratrol [35]. The authors observed that a moderate dose of resveratrol activated SIRT1 and induced liver kinase B1 deacetylation and AMPK activation, which led to increased mitochondrial biogenesis and function [35]. However, the protective effect of resveratrol against pancreatic cell damage in the MG-induced hyperglycaemic animal model via the SIRT1 and Nrf2 pathways has not been reported yet. Our previous study indicated that resveratrol could activate the ERK signaling pathway and enhance Nrf2 nuclear translocation and activation in RIN-m5F beta cells. One of the objectives of this study was to confirm the relation between resveratrol and Nrf2 in an animal model, since retinoic acid (RA) has been reported to suppress the activation of Nrf2 mediated by RA receptor-α. The results revealed that the effect of resveratrol was not abolished by RA treatment; therefore, there is a possibility that resveratrol can act not just via Nrf2.

We found that resveratrol markedly recovered the level of hepatic p-IRS-1 (Tyr) in MG-treated Balb/C mice and that this effect was abolished by RA treatment, suggesting that Nrf2 activation may have attenuated MG-induced insulin signaling pathway impairment (Figure 4). IHC analysis of β-cell insulin immunoreactivity (Figure 5) indicated that pancreatic insulin production was decreased by exposure to MG. Insulin immunoreactivity appeared to be elevated in animals treated with resveratrol and pioglitazone, but this recovery was impaired by RA. These findings suggested that Nrf2 played an important role in MG-induced loss of pancreatic insulin. Several potential mechanisms of Nrf2 phosphorylation by mitogen-activated protein kinase (MAPK) have been reported [48]. Therefore, we investigated pancreatic Nrf2 activation in MG-induced Balb/C mice. We found elevated pancreatic p-Nrf2 in mice that were co-administered MG and resveratrol; this effect was impaired by RA, while pioglitazone did not affect the pancreatic p-Nrf2 level in mice exposed to MG (Figure 6).

Abnormal cellular accumulation of MG invariably occurs in diabetes [49]. MG reduces glucose tolerance in rodents [50], suggesting that postprandial MG production in normoglycemic individuals could result in glucose intolerance. As a redox-dependent transcription factor, Nrf2 controls the expression of a number of genes, including those that encode stress response proteins and detoxifying enzymes. Therefore, the nuclear abundance of Nrf2 is tightly regulated by nuclear export and degradation processes. Nrf2 played an important role in improving glucose tolerance and insulin resistance in Nrf2-knockout mice fed a high-fat diet for 180 days [51]. Nrf2 activators protected against renal damage in streptozotocin-induced diabetes and activated the PKB signaling pathway to improve insulin sensitivity [52]. In agreement with these previous studies, our study indicated that resveratrol can at least partially activate the ERK signaling pathway, which was previously shown to enhance Nrf2 nuclear translocation and activation [39]. In addition, activation of Nrf2 by several compounds, including monascin, ankaflavin and dimerumic acid, elevated MG metabolism to alleviate diabetic damage *in vivo* [53,54,55,56]. Activation of Nrf2 also protected from oxidation of high-density lipoprotein cholesterol [57] and airway inflammation *in vivo* [58]. Resveratrol has been demonstrated to inhibit oxidative stress through Nrf2 activation [59,60]. We found that resveratrol effectively improved MG-induced effects on OGTT, ITT (Figure 1), hyperglycemia (Figure 2), inflammation (Figure 3) and pancreatic damage (Figure 5) and that these effects were associated with Nrf2 activation (Figure 6).

## 5. Conclusions

In conclusion, to study the significance of glycosylation-related stress on the pathology of diabetes, the effects of resveratrol were examined in a mouse model of diabetes. Resveratrol has been proposed to provide effective treatment of complications secondary to diabetes. This *in vivo* study found that resveratrol may also be useful for treating the insulin resistance found in diabetes.
